# Occupation-specific risk estimates for suicide and non-fatal self-harm from a Swedish cohort of male construction workers followed 1987–2018

**DOI:** 10.1136/oemed-2023-109246

**Published:** 2024-02-28

**Authors:** Kirsten Mehlig, Kjell Torén, Anthony D LaMontagne, Viktoria Wahlström, Jenny Nyberg, Margda Waern, Maria Åberg

**Affiliations:** 1 School of Public Health and Community Medicine, Institute of Medicine, University of Gothenburg, Gothenburg, Sweden; 2 Occupational and Environmental Medicine, School of Public Health and Community Medicine, Institute of Medicine, University of Gothenburg, Gothenburg, Sweden; 3 Institute for Health Transformation, Deakin University, Geelong, Victoria, Australia; 4 Department of Public Health and Clinical Medicine, Umeå Universitet, Umeå, Sweden; 5 Section for Clinical Neuroscience, Institute of Neuroscience and Physiology, University of Gothenburg, Gothenburg, Sweden; 6 Sahlgrenska University Hospital, Region Västra Götaland, Gothenburg, Sweden; 7 Department of Psychiatry and Neurochemistry, Institute of Neuroscience and Physiology, University of Gothenburg, Gothenburg, Sweden; 8 Department of Psychotic Disorders, Sahlgrenska University Hospital, Region Västra Götaland, Gothenburg, Sweden; 9 General Practice/Family Medicine, School of Public Health and Community Medicine, Institute of Medicine, University of Gothenburg, Gothenburg, Sweden; 10 Region Västra Götaland, Regionhälsan, Gothenburg, Sweden

**Keywords:** Mental Health, Epidemiology, Occupational Health, Construction Industry, Men

## Abstract

**Objectives:**

While suicidal behaviour has become less prevalent in non-manual workers in recent decades, rates have increased in manual workers. We aimed to identify occupations within the construction industry with excess risk of suicide and non-fatal self-harm.

**Methods:**

This cohort of Swedish construction workers comprises 389 132 individuals examined 1971–1993 and followed 1987–2018 using national hospital and cause of death registers. More than 200 job titles were merged into 22 occupational groups. For 296 891 men alive in 1987 and active in the construction sector, survival was calculated from baseline to first event of non-fatal self-harm or suicide and censored for emigration, long-term unemployment, disability pension, retirement, death from other causes or end of follow-up. HRs with 95% CIs were obtained from multiple Cox proportional hazard regression.

**Results:**

Overall, 1618 cases of suicide and 4774 events of non-fatal self-harm were registered. Self-harm before baseline was the single largest risk factor for suicide, HR 9.3 (95% CI 7.5 to 11.6). Compared with the overall mean, labourers and rock workers had excess risk for suicide, HR 1.4 (95% CI 1.1 to 1.7) and 1.5 (95% CI 1.0 to 2.3), respectively, while electricians, clerks and foremen had reduced risk. Labourers, concrete workers, sheet metal workers, painters, glaziers and the group ‘other construction workers’ were at increased risk for non-fatal self-harm. Almost all categories of manual workers were at increased risk for suicidal behaviour relative to clerks and foremen.

**Conclusions:**

Specific occupations within the construction sector were associated with excess risk for suicidal behaviour. Future studies should identify underlying risk factors to inform tailored interventions.

**WHAT IS ALREADY KNOWN ON THIS TOPIC**

- Construction workers have excess risk for suicidal behaviour compared with other employees but information is lacking which specific occupations are at greatest risk within the sector.

**WHAT THIS STUDY ADDS**

- Occupations with elevated risk of suicidal behaviour included labourers, rock workers, and sheet metal workers, with differential risks for suicide and non-fatal self-harm.

**HOW THIS STUDY MIGHT AFFECT RESEARCH, PRACTICE OR POLICY**

- The results show which occupations are at elevated risk for suicidal behaviour compared with the average risk for employees in the construction sector, and where prevention efforts should be intensified.

## Introduction

Suicide rates vary across countries and time periods, with presently highest rates in European countries and lowest rates reported in the Eastern Mediterranean,^1 2^ and temporal maxima during, for example, economic crises.^3^ There are also pronounced sex differences, as suicide is more common among men and non-fatal self-harm among women.^1 2 4^ Men tend to under-report mental ill health, more seldom seek professional help and use more lethal suicide methods than women.^5^ Psychiatric disorder and a history of suicide attempt are important predictors of suicidal behaviour.^1 6 7^ Recent reports from England and the USA show increasing trends in both suicide and non-fatal self-harm in the general population, indicating that non-fatal self-harm is both a risk factor and an important indicator for suicide risk.^8 9^ Large differences in suicide rates among employed men emphasise the importance of occupational aspects such as workplace conditions and job security.^10^ While early studies saw excess risk among men in professional occupations there was a reversal in trends, with increasing risk among manual workers in recent decades.^11 12^ A British study showed that suicide rates among manual workers were higher than among non-manual workers and professionals, and the rates among the former increased between 1979 and 2005, with labourers in building trades showing the second largest increase after coal miners.^12^ An Australian study demonstrated that occupational differences in suicide risk were exacerbated during financial crises; among men, the lower skilled workers such as labourers were most affected.^3^ Low skill level is also associated with low job control and job insecurity, both of which are associated with suicide in general working populations.^10 13–15^ In Sweden, the age-adjusted suicide rate for men was 15.8 per 100 000 in 2016, which was higher than the global mean of 13.7 per 100 000 in men of all ages.^16^ Swedish conscription data from 1985 to 2005 showed that there was a growing social divide in physical health among the conscripts.^17^ Subsequent economic recessions may have further exacerbated social differences in general health, disproportionally affecting manual workers.^3^

Workers within the construction sector comprise a high-risk group regarding suicidal behaviour, as they are exposed to several risk factors such as heavy physical workload, strict deadlines, dangerous working conditions and toxic substances.^1 18^ Specific psychological risk factors in this male-dominated occupation are a low awareness of mental health problems and a certain bullying culture particularly directed towards apprentices and newcomers to the industry.^19 20^ Compared with the general working population, excess risk of suicide in construction workers has been demonstrated previously,^11 12 21–23^ but a detailed analysis of specific occupations within the sector is lacking. Also, occupational risk for non-fatal self-harm is an area not often researched in spite of its importance not least for subsequent suicide; reasons for this research gap may include lack of reliable outcome information, in particular as most people who self-harm do not seek medical attention,^24^ which may be exacerbated among male construction workers for reasons given above. Episodes of self-harm among manual workers may also be misclassified as accidents, for instance, when heavy machinery is involved.^25^

Thus, the aim of this study was to identify specific occupations within the construction industry where workers are at excess risk for suicide and non-fatal self-harm. Analyses were based on data from a historical cohort of Swedish construction workers followed up to 32 years or until the end of active work life. Valid outcome measures were obtained through linkage to nationwide hospital and cause of death registers.^26^ Risk in individual occupations was compared with the average risk for suicidal behaviour in this sector, to describe the entire spectrum of risk estimates without singling out a specific reference occupation. The large size of almost 300 000 men allowed separate analyses of fatal and non-fatal self-harm in relation to 22 occupational groups.

## Methods

### Study population and definition of analytical sample

Following an agreement between employers and unions, Swedish construction workers were affiliated with the National Occupational Health Service (Bygghälsan) that offered free health examinations to employees in the construction sector on a regular basis between the mid-1960s and 1993. Although the programme was voluntary, about 80% of all workers participated in at least one examination.^27^ From 1971 on, data from medical examinations were entered into an electronic database. The current prospective study was based on data from 389 132 employees in the construction sector who participated in health examinations between 1971 and 1993, and were followed until 2018 using various national registers. Participants were re-examined up to 12 times after their first examination, with an average of three examinations in total. Observations from women who took part in the health examinations were excluded from this study because they constituted a minority (5%), and most were employed in administration (83% of all women). As most construction workers retired at age 65 during the study period, men who were aged 65 or above at their first health examination were excluded from the study. Further exclusions were made for men with missing occupational information. Because information on suicide was not available from the National Cause of Death register before 1987, the cohort was restricted to men alive and less than 65 years old in 1987. Lastly, we excluded all men who emigrated before 1987. The final analytical sample consisted of 296 891 male construction workers aged 16–64 (online supplemental table S1). For all participants, we had information on the year of birth as well as the region where the baseline examination was performed (14 regions from all over Sweden). It is noted that data were not collected for research purposes but to monitor individuals’ health, and in contrast to health parameters, other information such as occupation was not always noted. Because the lack of occupational information can be assumed to be missing at random, the listwise deletion of observations with missing exposure values is not likely to cause bias in the association analyses presented below.

10.1136/oemed-2023-109246.supp1 Supplementary data

**Table 1:** Cohort description at baseline† and at last health examination

	Participants	Age	Participants at last health visit
Occupation	N (%)	Mean (SD)	N (%)
Road construction worker	3652 (1.2)	36.1 (10.8)	3671 (1.2)
Rock worker	2622 (0.9)	38.9 (10.4)	2404 (0.8)
Labourer	10 215 (3.4)	36.2 (11.6)	10 287 (3.5)
Concrete worker	28 052 (9.5)	37.5 (12.4)	27 079 (9.1)
Carpenter	62 628 (21.1)	32.7 (11.2)	61 795 (20.8)
Bricklayer	8514 (2.9)	35.6 (12.2)	8347 (2.8)
Floor layer	5183 (1.8)	32.7 (10.5)	5144 (1.7)
Glazier	2506 (0.8)	32.2 (10.7)	2482 (0.8)
Insulation worker	2679 (0.9)	32.8 (11.0)	2534 (0.9)
Sheet metal worker	11 353 (3.8)	31.2 (10.4)	11 009 (3.7)
Roofer	1289 (0.4)	34.5 (10.0)	1331 (0.5)
Pipe fitter, plumber	22 131 (7.5)	34.0 (11.6)	21 296 (7.2)
Painter	21 088 (7.1)	32.6 (11.3)	21 013 (7.1)
Machine operator	9819 (3.3)	37.2 (10.3)	9566 (3.2)
Crane operator	2886 (1.0)	39.9 (9.4)	2891 (1.0)
Driver	3998 (1.4)	38.6 (10.8)	3837 (1.3)
Refrigeration mechanic	1296 (0.4)	32.5 (10.0)	1288 (0.4)
Reparation mechanic	2594 (0.9)	36.9 (11.1)	2497 (0.8)
Electrician	34 712 (11.7)	31.3 (10.4)	33 673 (11.3)
Other construction work*	17 724 (6.0)	32.6 (12.5)	18 922 (6.4)
Foreman	29 002 (9.8)	38.4 (10.1)	32 238 (10.9)
Clerk, employee	12 948 (4.4)	41.0 (9.8)	13 587 (4.6)
Total	296 891 (100)	37.2 (13.4)	296 891 (100)

*Scaffold builder, welder, blacksmith, etc.

†Most recent health examination ≤1987 or directly after.

### Exposure

More than 200 job titles within the construction sector were registered between 1971 and 1993.^28^ These were combined into 22 groups of comparable task and skill level as proposed by technical experts from industry and unions, and included 19 groups of manual workers, clerks, foremen, as well as other workers within the construction sector (table 1). Detailed descriptions of the 19 manual work groups have been published previously.^29^ In this study, the term ‘labourers’ is used to denote ground preparatory workers. Examples of job titles in the ‘other construction workers’ category are scaffold builders, welders, and blacksmiths. Baseline occupation was defined as the occupation reported at the last examination before 1987, or, for those who entered the cohort after 1986, the occupation reported at the first examination. To account for the effect of switching among occupational groups, we also registered the occupation reported at the last health visit.

### Outcomes and censoring information

Information on suicide deaths was obtained from the National Cause of Death Register and the National Hospital Register provided data on non-fatal self-harm. Self-harm is defined here as any type of self-injurious behaviour, including both suicide attempts and non-suicidal self-injuries.^1^ Death by suicide and first episode of non-fatal self-harm were the main endpoints in this study and included events both with (International Classification of Diseases (ICD)-8/9: E95; ICD-10: X6, X7, X80–X84, Y870) and without (ICD-8/9: E98; ICD-10: Y1 Y2 Y30–Y34 Y872) determined intent. As some cases registered as self-harm of undetermined intent may have been accidental, we then carried out separate analyses after exclusion of cases with undetermined intent. Regarding non-fatal self-harm, we also distinguished between episodes before baseline examination and incident events. Observations were censored in connection with emigration due to lack of prospective information. Observations were also censored at the first time of unemployment and disability pension as both phenomena are associated with suicide^1^ and can be expected to vary across occupations. Information on emigration and disability pension was obtained from the longitudinal integrated database for Health Insurance and Labour Market Studies (LISA) covering the years 1990–2018. The number of days on unemployment benefit was available from 1992 onwards and long-term unemployment was defined as 1 year or more. The LISA register includes detailed data on health and social insurance and is updated yearly (Statistics Sweden SCB).

### Statistical methods

To assess how occupation reported at baseline was associated with self-harm before baseline we used logistic regression and reported results as ORs with 95% CI. The prospective associations between 22 occupational groups and risk of suicide or non-fatal self-harm were examined using Cox proportional hazard models. Baseline was defined as 1 January 1987, or the date of the first health examination after that date. Observations were censored at first emigration after baseline, long-term unemployment, receipt of disability pension, age 65, death from other causes or end of 2018, whatever occurred first. Survival from baseline until the date of suicide, first record of non-fatal self-harm or censoring was calculated. For endpoints (suicide and non-fatal self-harm) with determined intent survival was calculated until the event of interest or censoring irrespective of previous events lacking determined intent. Men with a record of self-harm before baseline were excluded from prospective analyses of non-fatal self-harm to reduce the risk of previous mental health problems influencing the choice of occupation (reverse causation). Models for suicide were adjusted for self-harm before baseline, to account for previous mental health problems. As suggested by Stack,^30^ regression models were adjusted for demographic variables, that is, age (with quadratic and cubic terms), region and year of occupational report. The proportional hazard assumption was tested and confirmed graphically for all occupational groups. Because endpoints were generally rare, we used penalised likelihood estimation to reduce the small sample bias in maximum likelihood estimation.^31^ Results were given in terms of HRs and CIs. As there is no natural reference category, we used deviation from means coding to calculate the risk for individual occupations compared with the overall risk for suicide or self-harm in the sample. This is done by obtaining linear combinations of occupation-specific beta-values such that their sum is zero, and the mean risk is given by the intercept in the logistic regression model and the baseline hazard in the survival model, respectively.^32^ Furthermore, we examined the association between the occupation reported at the last health examination and suicidal behaviour, again excluding all men with episodes of self-harm before the last health visit from the analysis of non-fatal self-harm. This was done to assess whether associations with the most recent occupation differed from results for baseline occupational exposure. Effect modification by self-harm before baseline was examined by adding product terms for self-harm before baseline and occupations to the model for suicide, and the p value for an overall F-test comparing models with and without interactions terms was given. Statistical analyses were performed by using SAS V.9.4 and Matlab (R2016b; The MathWorks). The significance level was set at 0.05 (two-sided tests).

## Results

### Cohort description

Table 1 shows the number and mean age of workers, overall and by occupational group. At baseline, the mean age of construction workers was 37.2 years with differences between occupational groups. Among the workers with at least two examinations (64%), the mean number of changes between occupational categories was 0.3, range=0–7, and 77% did not change category. The right column of table 1 shows the distribution of occupational groups reported at the last health examination before the health programme ended in 1993. The prevalence of manual occupations remained stable, but the proportion of foremen increased by 1.1%.

### Associations with self-harm before baseline

Episodes of self-harm before baseline were recorded for 2072 participants (0.3%). Several occupations showed excess risk for self-harm prior to baseline, that is, insulation workers, roofers, rock workers, labourers, crane operators, glaziers, floor layers, other construction workers and concrete workers, in order of decreasing risk compared with mean risk (table 2). Carpenters, electricians, clerks and foremen were less likely to have a history of self-harm compared with overall risk. To limit the potential for confounding, men with self-harm before baseline were excluded from the prospective analysis of non-fatal self-harm. For the same reason, the analysis of suicide was adjusted for self-harm before baseline.

**Table 2:** Occupation at baseline and odds for self-harm prior to baseline (n=2072/296 891)

Baseline occupation	No of cases (%)	OR (95% CI)‡
Road construction worker	29 (0.8)	1.02 (0.72 to 1.45)
Rock worker	33 (1.3)	1.52 (1.09 to 2.12)*
Labourer	124 (1.2)	1.49 (1.24 to 1.79)***
Concrete worker	295 (1.1)	1.29 (1.14 to 1.47)***
Carpenter	386 (0.6)	0.80 (0.71 to 0.90)***
Bricklayer	78 (0.9)	1.16 (0.93 to 1.44)
Floor layer	57 (1.1)	1.43 (1.11 to 1.85)**
Glazier	29 (1.2)	1.46 (1.03, to 2.08)*
Insulation worker	38 (1.4)	1.87 (1.37 to 2.56)***
Sheet metal worker	90 (0.8)	1.05 (0.85 to 1.29)
Roofer	19 (1.5)	1.84 (1.19 to 2.83)**
Pipe fitter, plumber	170 (0.8)	0.97 (0.83 to 1.14)
Painter	161 (0.8)	0.96 (0.81 to 1.13)
Machine operator	63 (0.6)	0.82 (0.64 to 1.05)
Crane operator	37 (1.3)	1.46 (1.07 to 2.00)*
Driver	21 (0.5)	0.69 (0.46 to 1.04)
Refrigeration mechanic	10 (0.8)	0.98 (0.54 to 1.75)
Reparation mechanic	16 (0.6)	0.84 (0.53 to 1.34)
Electrician	142 (0.4)	0.52 (0.44 to 0.61)***
Other construction work†	162 (0.9)	1.39 (1.18 to 1.64)***
Foreman	78 (0.3)	0.31 (0.25 to 0.39)***
Clerk, employee	34 (0.3)	0.33 (0.24 to 0.46)***

*p<0.05, **p<0.01, ***p<0.001.

†Scaffold builder, welder, blacksmith, etc.

‡Logistic regression adjusted for age, year, and region (ref=overall mean).

### Risk for suicide and self-harm among construction workers

Overall, 2624 cases of suicide and 7187 cases of non-fatal self-harm were recorded between baseline and end of follow-up in 2018. Censoring at age 65, emigration after baseline, unemployment and disability pension reduced the numbers to 1618 suicides and 4774 incident cases of non-fatal self-harm. Mean age at suicide was 46.7 years, and the corresponding figure for first episode non-fatal self-harm in men with no self-harm before baseline was 46.4 years. Average follow-up was 20.8 years for suicide (11.5 years among cases of suicide), and 20.6 years for non-fatal self-harm excluding observations with self-harm before baseline (15.5 years among cases of non-fatal self-harm).

**Figure 1:**
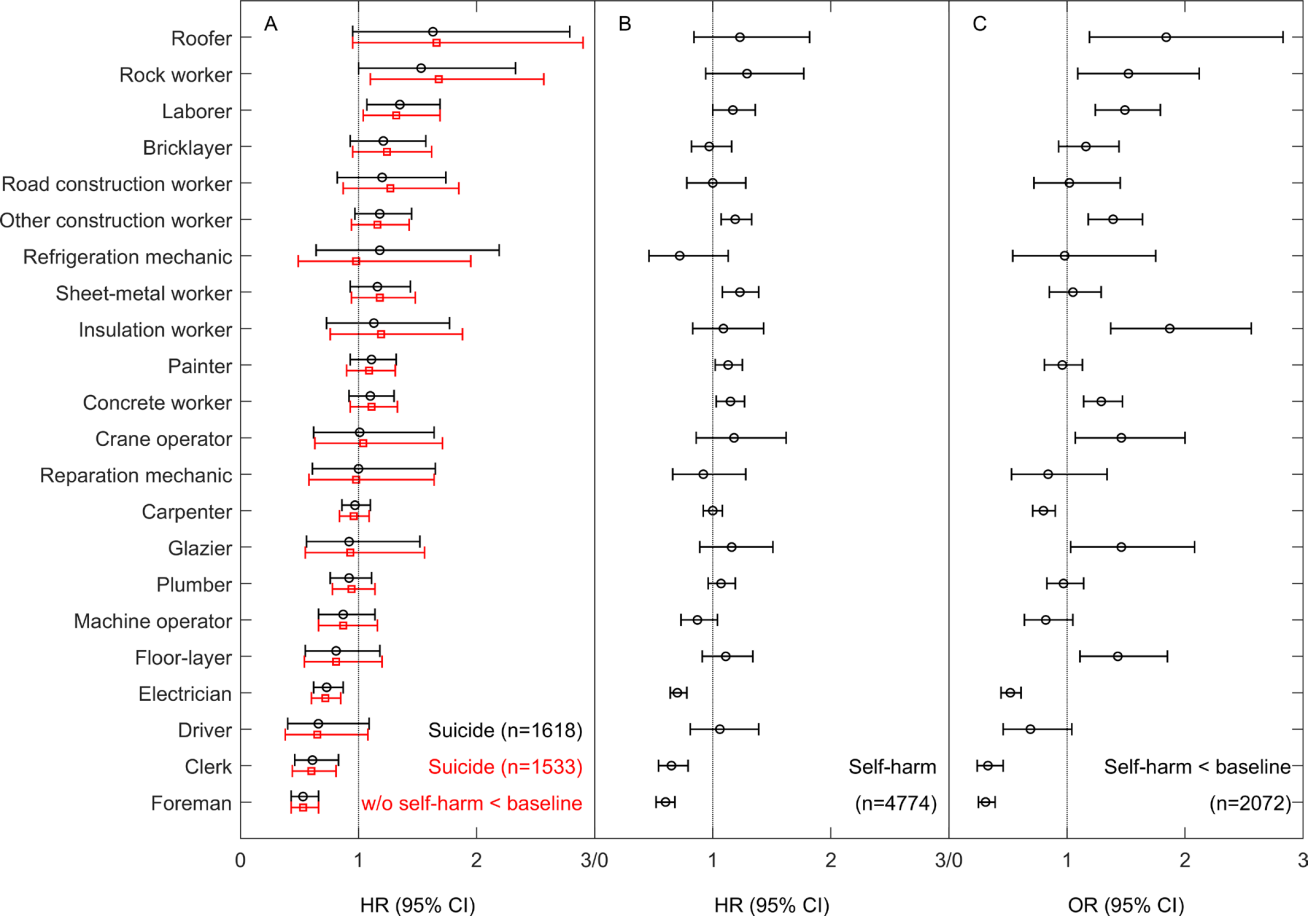
Risk for suicide and non-fatal self-harm for manual and non-manual occupations within the construction sector (HRs from prospective analyses of suicide and non-fatal self-harm, ORs from cross-sectional analyses of self-harm before baseline; ref = overall mean risk).

Table 3 presents the associations between occupational categories and suicide and incident non-fatal self-harm after baseline, overall and restricted to endpoints with determined intent. Episodes of self-harm before baseline were a strong risk factor for suicide, HR=9.3 (95% CI 7.5 to 11.6). The HR remained high after censoring for suicide with undetermined intent, HR=8.0 (95% CI 6.2 to 10.5). Labourers, rock workers and other construction workers showed elevated risk for suicide compared with the overall mean, and these associations were particularly pronounced when deaths of undetermined intent were censored. The occupation-specific associations were not confounded by self-harm before baseline (not shown) nor were they modified by the latter (p value for interaction between occupation and self-harm before baseline in relation to suicide=0.7). Figure 1A shows that the occupation-specific risk estimates for suicide were hardly affected on exclusion of workers with previous self-harm. Sheet metal workers, other construction workers, painters, labourers and concrete workers showed elevated risk for non-fatal self-harm. Risk estimates were enhanced with respect to non-fatal self-harm with determined intent but only for sheet metal workers, painters and other construction workers. Non-manual workers (clerks, foremen) as well as electricians had lower risks for suicide and self-harm compared with the overall mean. Figure 1 shows the consistency of occupation-specific risk across endpoints.

**Table 3:** Occupation at baseline and risk for suicide and non-fatal self-harm

	Suicide (n=296 891)		Non-fatal self-harm (n=294 819)	
	All cases (n=1618)	With known intent (n=1309)	All cases (n=4774)	With known intent (n=2169)
Occupation	HR (95% CI)†‡	HR (95% CI)†‡	HR (95% CI)†	HR (95% CI)†
Road construction worker	1.20 (0.82 to 1.74)	1.14 (0.75 to 1.74)	1.00 (0.78 to 1.28)	1.20 (0.85 to 1.69)
Rock worker	1.53 (1.00 to 2.33)*	1.85 (1.20 to 2.86)**	1.29 (0.94 to 1.77)	1.15 (0.72 to 1.84)
Labourer	1.35 (1.07 to 1.69)*	1.31 (1.01 to 1.70)	1.17 (1.00 to 1.36)*	1.15 (0.92 to 1.44)
Concrete worker	1.10 (0.92 to 1.30)	1.00 (0.82 to 1.22)	1.15 (1.03 to 1.27)*	1.11 (0.95 to 1.29)
Carpenter	0.97 (0.86 to 1.10)	0.98 (0.85 to 1.13)	1.00 (0.92 to 1.08)	0.91 (0.81 to 1.02)
Bricklayer	1.21 (0.93 to 1.57)	1.07 (0.78 to 1.46)	0.97 (0.82 to 1.16)	1.20 (0.95 to 1.51)
Floor layer	0.81 (0.55 to 1.18)	0.85 (0.56 to 1.28)	1.11 (0.91 to 1.34)	1.08 (0.81 to 1.44)
Glazier	0.92 (0.56 to 1.52)	0.84 (0.47 to 1.50)	1.16 (0.89 to 1.51)	1.40 (0.98 to 2.00)
Insulation worker	1.13 (0.73 to 1.77)	1.18 (0.73 to 1.92)	1.09 (0.83 to 1.43)	1.19 (0.82 to 1.74)
Sheet metal worker	1.16 (0.93 to 1.44)	1.10 (0.85 to 1.41)	1.23 (1.08 to 1.39)**	1.26 (1.05 to 1.51)*
Roofer	1.63 (0.95 to 2.79)	1.72 (0.96 to 3.09)	1.23 (0.84 to 1.82)	1.14 (0.64 to 2.05)
Pipe fitter, plumber	0.92 (0.76 to 1.11)	0.91 (0.74 to 1.12)	1.07 (0.96 to 1.19)	1.11 (0.95 to 1.29)
Painter	1.11 (0.93 to 1.32)	1.14 (0.93 to 1.39)	1.13 (1.02 to 1.25)*	1.39 (1.21 to 1.60)***
Machine operator	0.87 (0.66 to 1.14)	0.85 (0.62 to 1.16)	0.87 (0.73 to 1.04)	0.77 (0.59 to 1.02)
Crane operator	1.01 (0.62 to 1.64)	0.88 (0.49 to1.58)	1.18 (0.86 to 1.62)	1.20 (0.77 to 1.87)
Driver	0.66 (0.40 to 1.09)	0.71 (0.42 to 1.22)	1.06 (0.81 to 1.39)	1.07 (0.74 to 1.57)
Refrigeration mechanic	1.18 (0.64 to 2.19)	1.16 (0.58 to 2.31)	0.72 (0.46 to 1.13)	0.81 (0.44 to 1.49)
Reparation mechanic	1.00 (0.61 to 1.65)	0.90 (0.50 to 1.62)	0.92 (0.66 to 1.28)	0.85 (0.51 to 1.39)
Electrician	0.73 (0.62 to 0.87)***	0.78 (0.65 to 0.94)**	0.70 (0.64 to 0.78)***	0.55 (0.47 to 0.65)***
Other construction work§	1.18 (0.97 to 1.45)	1.35 (1.09 to 1.66)**	1.19 (1.07 to 1.33)**	1.24 (1.06 to 1.46)**
Foreman	0.53 (0.43 to 0.66)***	0.57 (0.45 to 0.72)***	0.60 (0.52 to 0.68)***	0.50 (0.41 to 0.61)***
Clerk, employee	0.61 (0.46 to 0.83)**	0.64 (0.46 to 0.89)**	0.65 (0.54 to 0.79)***	0.56 (0.41 to 0.75)***

*p<0.05, **p<0.01, ***p<0.001.

†Cox proportional hazard regression adjusted for age, year and region (ref=overall mean).

‡Further adjusted for self-harm before baseline

§Scaffold builder, welder, blacksmith, etc.

**Figure 2:**
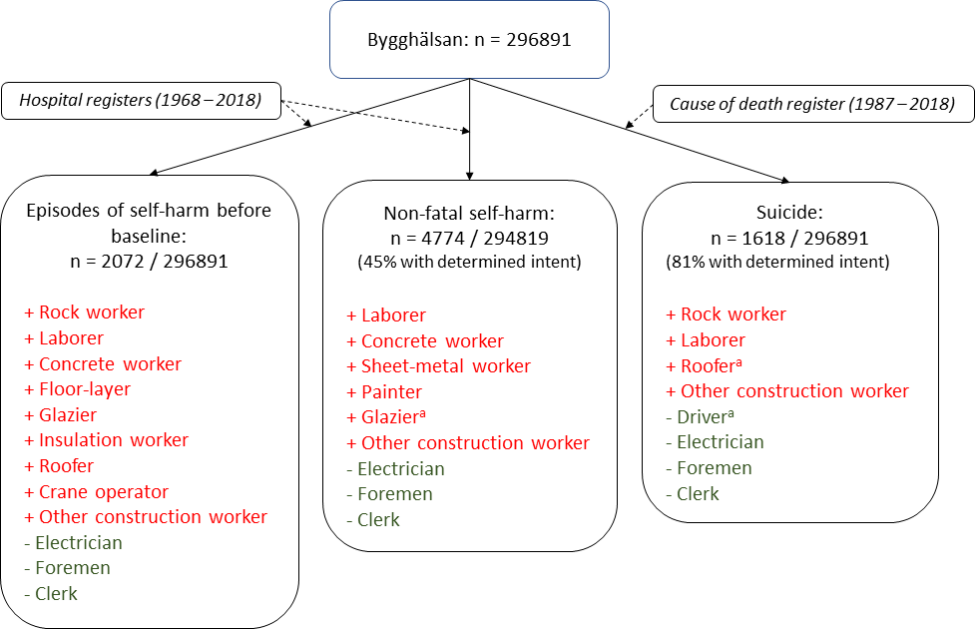
Summary of significant occupation-specific associations with suicidal behaviours (occupations with excess risk in red, those with reduced risk in green; ref=mean risk for suicidal behaviour). ^a^Only observed for occupations reported at last health examination.

Analyses using occupation at the last health examination, that is, the most recent occupation known in relation to outcome hardly changed the associations reported for baseline exposure, primarily due to the small number of changes in occupation recorded during 1971–1993 (online supplemental table S2). Roofers showed >60% excess risk for suicide compared with the overall mean, a result that was statistically significant only when considering occupations at last health visit. Reduced suicide risk for drivers was observed with respect to occupation reported at last visit only. Similarly, 45% excess risk for glaziers with respect to non-fatal self-harm was significant only if reported at last health visit. An overview of occupation-specific associations with suicidal endpoints is given in figure 2.

## Discussion

This study aimed to identify specific occupations within the construction sector with excess risk of suicidal behaviour compared with the mean risk in the entire cohort. Rock workers, labourers and roofers showed excess risk for suicide, while the higher skilled and higher status occupations of electricians, clerks and foremen were at reduced risk. Adverse associations increased in magnitude for rock workers and the group ‘other construction workers’ when analyses were restricted to suicide with determined intent. With the exception of labourers and the ‘other construction worker’ group the pattern differed with respect to non-fatal self-harm where excess risk was displayed by sheet metal workers, painters, glaziers and concrete workers.

This study showed that workers in specific occupational groups were at increased risk for different types of suicidal behaviour. Below, we offer possible explanations for the observed associations, which support the plausibility of results and may guide future studies and interventions. Compared with the overall mean electricians were the only manual workers who showed significantly lower risk for all types of suicidal behaviour, which may be related to higher skill level, better pay and better control over work tasks compared with other manual workers. In contrast, several occupations showed excess risk for specific endpoints, namely labourers, rock workers, and roofers for suicide, and sheet metal workers, painters, glaziers, and concrete workers for self-harm. A risk factor common to labourers, rock workers and roofers is the heavy physical labour that may lead to poor physical health including disability and chronic pain, which also affect mental health.^33^ Previous findings from the same cohort confirm that these workers were more likely to receive disability pension compared with other construction workers.^27 28^ Labourers and rock workers were also among the oldest in this cohort, and thus more likely to be affected by the deterioration of physical health. Road construction work is characterised by heavy physical labour as well, yet it was not associated with any suicidal behaviour. A reason may be that the work is very intense during the ice-free season and with long holidays and a possibility for recovery during wintertime.

While excess risk for suicide among labourers and roofers has been shown previously,^12^ our study provides further occupation-specific results for non-fatal self-harm. A study of 16-year old children from the British ALSPAC cohort showed differential risk factors for self-harm with and without suicidal intent, that is, higher values of IQ and maternal education for non-suicidal self-harm, and, among others, lower values of IQ and socioeconomic position for self-harm with suicidal intent.^4^ Although populations and outcome measures differ we note that the occupations associated with excess risk for suicide in our study (labourers, rock workers, roofers) were previously classified as low-skilled occupations while occupations associated with self-harm (sheet metal workers, painters, glaziers, concrete workers) were at higher skill level.^28^ Since a positive correlation between IQ and professional skill level is plausible, our findings may provide further evidence for different risk factors behind the two kinds of suicidal behaviour including personality traits beyond IQ. Last, occupation-specific associations were largely consistent across non-fatal endpoints except for insulation workers, crane operators, glaziers and floor layers, who showed associations with self-harm before baseline, but no prospective associations. It is possible that the exclusion of individuals with episodes of self-harm before baseline reduced the possibility to capture prospective associations with non-fatal self-harm.

### Strengths and limitations

The large, homogeneous cohort of construction workers is an important strength of the study that speaks for similar sociocultural attitudes towards suicide.^1^ The prospective design with long follow-up is a strength of the study, but the lack of occupational information after 1993 is a limitation as more recent occupations may show a stronger link with suicidal behaviour, simply due to temporal proximity. We showed that using occupational information from the last health examination hardly changed the results, mainly because most workers kept their occupation while employed in the construction sector, but it is not clear whether this holds after 1993. Second, the lack of information from the cause of death registry before 1987 is a limitation that forced us to exclude observations from 4548 men, who died before 1987. This exclusion may have biased our results towards the null, in particular as the men, who died before 1987 were more likely to belong to risk groups, that is, rock workers, concrete workers, or workers from among the group of ‘other construction workers’, compared with the survivors (not shown). Last, these analyses are essentially descriptive, looking for subgroups with excess risk rather than causal factors. The observed associations could be due to selection into occupations or to the nature of and exposure in those occupations, or a combination of both. Even if occupation-specific risks are plausible, the lack of information on substance use and other mental disorders apart from a history of non-fatal self-harm is a limitation of this study as these risk factors may differ across occupational groups.^18 34^ However, a Danish study reported few occupational differences in suicide risk among people who suffered from psychiatric illness,^35^ a finding that was paralleled in our study regarding self-harm before baseline.

### Conclusions and implications for policy and practice

In this study, we identified specific high-risk occupations within the construction sector that should be prioritised for suicide prevention. Our results are expected to be relevant today and across countries as tasks and conditions for construction work hardly vary, partly due to persistent economic difficulties that hamper the improvement of unfavourable work environments. Further research is needed to understand risk and protective factors in specific groups, to tailor interventions accordingly.

## Data Availability

Data are available on reasonable request. Data will be made available on reasonable request following a decision by the steering committee.
