# Supplementary material for: Occupation-specific risk estimates for suicide and non-fatal self-harm from a Swedish cohort of male construction workers followed 1987–2018
Source: Occup Environ Med. 2024 Feb 28;81(3):142–9. doi: 10.1136/oemed-2023-109246 (PMC10958292; doi:10.1136/oemed-2023-109246)
Supplement: Supplementary data [file oemed-2023-109246supp001.pdf]

SUPPLEMENT

Table S1: Definition of analytic sample

N = 389 132	Exclusions
	Women (n = 19 418)
N = 369 714	
	Age at baseline examination > 64 years (n = 6843)
N = 362 871	
	Occupation not reported at any health examination (n = 18 436)
N = 344 435	
	Age > 64 years before 1987 (n = 40 963)
N = 303 472	
	Death before 1987 (n = 4548)
N = 298 924	
	Emigration between last health examination and 1987 (n = 2033)
N = 296 891	

Table S2: Occupation registered at last health examination and risk for incident suicidal behavior

	Suicide (n = 296 352)		Non-fatal self-harm (n = 294 156)	
	All cases (n = 1618)	With known intent (n = 1309)	All cases (n = 4647)	With known intent (n = 2086)
Occupation at last health visit	HR (95% CI) [ab]	HR (95% CI) [ab]	HR (95% CI) [a]	HR (95% CI) [a]
Road construction worker	1.31 (0.91, 1.90)	1.19 (0.78, 1.82)	1.02 (0.80, 1.31)	1.28 (0.90, 1.81)
Rock worker	1.48 (0.95, 2.30)	1.77 (1.12, 2.80)*	1.28 (0.92, 1.78)	1.14 (0.69, 1.88)
Laborer	1.43 (1.15, 1.80)**	1.40 (1.09, 1.80)**	1.08 (0.92, 1.27)	1.07 (0.85, 1.35)
Concrete worker	1.10 (0.93, 1.31)	1.00 (0.82, 1.22)	1.19 (1.07, 1.32)**	1.23 (1.05, 1.43)**
Carpenter	0.99 (0.87, 1.13)	0.99 (0.86, 1.14)	1.00 (0.93, 1.08)	0.94 (0.84, 1.06)
Bricklayer	1.27 (0.98, 1.65)	1.13 (0.83, 1.54)	0.89 (0.74, 1.07)	1.10 (0.86, 1.41)
Floor-layer	0.83 (0.57, 1.21)	0.86 (0.57, 1.30)	1.10 (0.90, 1.33)	1.08 (0.80, 1.45)
Glazier	0.95 (0.58, 1.56)	0.85 (0.47, 1.53)	1.15 (0.87, 1.51)	1.45 (1.02, 2.07)*
Insulation worker	1.07 (0.67, 1.71)	1.08 (0.64, 1.82)	0.97 (0.72, 1.31)	0.99 (0.65, 1.53)
Sheet-metal worker	1.18 (0.94, 1.47)	1.08 (0.84, 1.40)	1.23 (1.08, 1.40)**	1.25 (1.03, 1.51)*
Roofer	1.69 (1.01, 2.84)*	1.63 (0.91, 2.94)	1.41 (0.99, 2.01)	1.25 (0.71, 2.18)
Pipe fitter, plumber	0.98 (0.81, 1.18)	0.97 (0.79, 1.19)	1.08 (0.97, 1.21)	1.15 (0.98, 1.34)
Painter	1.16 (0.97, 1.38)	1.18 (0.97, 1.43)	1.14 (1.03, 1.27)*	1.41 (1.22, 1.62)***
Machine operator	0.90 (0.69, 1.18)	0.86 (0.63, 1.17)	0.90 (0.76, 1.08)	0.85 (0.65, 1.11)
Crane operator	1.15 (0.73, 1.82)	1.12 (0.67, 1.88)	1.11 (0.80, 1.53)	1.12 (0.70, 1.79)
Driver	0.43 (0.23, 0.80)**	0.48 (0.25, 0.93)*	1.06 (0.81, 1.39)	1.03 (0.69, 1.52)
Refrigeration mechanic	1.49 (0.85, 2.60)	1.50 (0.81, 2.78)	0.88 (0.58, 1.33)	0.86 (0.47, 1.60)
Reparation mechanic	0.81 (0.46, 1.41)	0.83 (0.45, 1.53)	0.86 (0.61, 1.22)	0.82 (0.49, 1.37)
Electrician	0.77 (0.65, 0.91)**	0.81 (0.68, 0.97)	0.71 (0.64, 0.78)***	0.57 (0.49, 0.67)***
Other construction work [c]	1.16 (0.96, 1.41)	1.26 (1.02, 1.55)*	1.13 (1.01, 1.26)*	1.20 (1.01, 1.41)*
Foreman	0.57 (0.47, 0.70)***	0.61 (0.49, 0.75)***	0.64 (0.57, 0.72)***	0.49 (0.40, 0.60)***
Clerk, employee	0.50 (0.37, 0.68)***	0.53 (0.38, 0.74)***	0.63 (0.52, 0.76)***	0.56 (0.42, 0.75)***

[a] Cox proportional hazard regression adjusted for age, year, and region (ref=overall mean)
[b] Further adjusted for self-harm before baseline
[c] Scaffold builder, welder, blacksmith etc.
* p <.05, ** p <.01, *** p <.001
